# A FOLLOW UP STUDY ON REVISED NATIONAL TUBERCULOSIS CONTROL PROGRAMME (RNTCP): RESULTS FROM A SINGLE CENTRE STUDY

**DOI:** 10.4103/0970-2113.45277

**Published:** 2008

**Authors:** R. Prasad, S. K. Verma, P. Shrivastava, S. Kant, R. A. S. Kushwaha, S. Kumar

**Affiliations:** Department of Pulmonary Medicine, CSMMU, UP, Lucknow, India

**Keywords:** RNTCP India, Relapse, Tuberculosis

## Abstract

**Objective:**

To know the follow up status of tuberculosis patients after 1 year of completion of treatment in RNTCP.

**Materials & Methods:**

Those patients of tuberculosis, who were registered from June 2003 to June 2004 at DOTS centre of our institution, were followed up at their homes after one year of completion of treatment between August 2005 and August 2006, with the help of health visitor. Patients were followed up clinically and symptomatic patients were also followed radiologically as well as bacteriologicalty. Results of the study were recorded in terms of number of patients remained asymptomatic, number of patients relapsed and number of patients died.

**Results:**

Out of 237 patients registered, 8 patients died during treatment period, 12 patients defaulted the treatment, the number of failures was 5 and 212 patients were declared cured/treatment completed. Out of these 212 patients 60 were unavailable for interview due to various reasons. The study observed that out of a total of 152 patients interviewed, 137 patients (90.1%) of remained asymptomatic, 11 patients (7.2%) had relapsed and 4 patients (2.6%) died during follow up.

**Conclusion:**

The study observed that majority of patients (90.1%) re-mained asymptomatic after the completion of one year of treatment under DOTS.

## INTRODUCTION

Tuberculosis kills more adults in India than any other infectious disease. Almost two people die of it in our country, every three minutes^1^. Unfortunately, despite the existence of the National Tuberculosis Control Programme from 1962 till 1992, the desired results have not been achieved. Revised National Tuberculosis Control Programme (RNTCP) came into existence by formulating and adopting the internationally recommended Directly Observed Treatment Short course(DOTS) strategy as the most systemic and cost effective approach to revitalize the TB control programme in India. To achieve the goal the first objective is to achieve and maintain - a cure rate of at least 85% among newly detected infectious (new sputum smear positive) cases and to achieve and maintain detection of at least 70% of such cases in the population. Treatment success rate has been increased from 25% to 86% from 1998 to 2004. Death rate has been brought down seven-folds, from 29% to 4%^1^,^2^. Despite high cure rates, several queries and doubts have been raised by the clinicians either from government /private sector about the effectiveness of RNTCP regimens, inadequate diagnosis, method of administration, wrong categorization and increased rate of multi drug resistant tuberculosis. Despite 8 years of its existence, to the best of our knowledge, not a single study was done on follow up after one year of completion of treatment in all three categories simultaneously.

Till date very few follow up studies have been done to prove the efficacy of Revised National Tuberculosis Control Programme of India and not a single study was done on follow up after one year of completion of treatment in all three categories simultaneously. The present study was done to know the follow-up status of tuberculosis patients, treated under RNTCP of India, who were followed after 1 year of completion of treatment between August 2005 and August 2006.

## MATERIALS AND METHODS

A total of 237(Cat I: 114, Cat II: 67 Cat III: 56) patients of tuberculosis, registered between June 2003 and June 2004 at DOTS centre situated at Department of Pulmonary Medicine, King George's Medical University (KGMU), Lucknow, a tertiary care centre and a large teaching institution, were included in the study. Information about the name, age, sex, address, initial sputum smear result, during and at the end of their treatment, treatment category, date of start of treatment and outcome were collected from the Tuberculosis register (TB Register) maintained at the DOTS centre. All the patients along with their addresses were approached, door-to-door, at their homes with the help of health visitor. Written informed consent was taken from all the patients, found after 1 year, in order to participate in the study. Patients were evaluated clinically in a pre¬designed Performa. All the symptomatic patients were subjected to Chest x-ray PA view, sputum smear test for Acid Fast Bacilli on three occasions (early morning sample) and culture for Mycobacterium tuberculosis. Their detailed radiological evaluation was done by expert chest physician at our department.

## RESULTS

During the period from June 2003 to June 2004, a total of 237 patients were registered for treatment in all categories (Cat I: 114, Cat II: 67 Cat III: 56). Of these, 129 (54.4%) were males and 69(53%) patients were in 11-30 years age group and 108 (45.6%) were females, 80(74%) in 11-30 years age group. (Table-I)

**Table I:** Distribution of patients : age and sex wise.

Age group (Years)	Male (n=129)		Female (n = 180)
	No. of Patients	Percentage (%)	No. of Patients	Percentage (%)
0–10	04	3.10	01	09.03
11–20	32	27.81	40	37.04
21–30	37	28.68	40	37.04
31–40	28	21.71	15	13.89
41–50	18	13.95	07	06.48
51–60	04	03.10	03	02.78
61–70	06	04.65	01	00.93
71–80	00	–	01	00.93

Overall Treatment success (cured/treatment completed) in study group were 89.4 % (212/237). Treatment success among Cat-I was 89.5 %(102/114), among Cat-II was 86.6 %(58/67) and among Cat-III was 92.9 %(52/56). (Table II)

**Table II:** Outcome Patients after completion of DOTS (n= 237)

Category	Patient Registered	Cured	Treatment Completed	Defaulter	Failure	Died
I	114	070	032	006	002	003
II	067	040	018	003	002	005
III	056	042	010	003	001	000

Total	237	152	060	012	005	008

Information was available for 152(71.7%) patients only whereas 34(16%) patients had migrated to some other place. 26(12.3%) patients could not be traced due to improper address over record register and 8 died during treatment (Figure-I). Status of total 152patients revealed that 137(90.1%) patients had no complaints., 4(2.6%) patients who took treatment died during study period, one patient was killed in an accident and rest three died due to natural causes. At the time of follow-up 11(7.2%) patients had relapsed, 5 in CAT-I, 5 in CAT- II and 1 in CAT-III.

**Figure:**
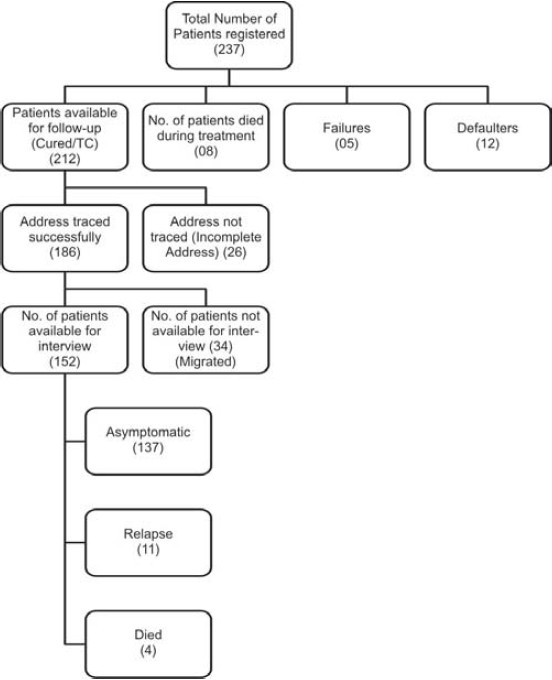


## DISCUSSION

Perhaps the greatest strength of the RNTCP is the new recording and reporting system, which enables periodic evaluation of the programme^3^. The cohort of patients diagnosed and put on treatment from June 2003 to June 2004 were followed up between August 2005 & August 2006 after lapse of varying periods of observation. Our study observed the relapse rate of 7.2% after one year while one study done in Mumbai, India and other in South Africa observed the relapse rate of 3.8% and 5% respectively^2^,^3^. Our study showed a similar figure in comparison to controlled clinical trials in which patients were followed up regularly for 2 years or more have shown that the frequency of relapse is around 3-7% with standardized short-course chemotherapy^5^. Various studies, done to evaluate the efficacy of thrice weekly regimen of short course chemotherapy also revealed a relapse rate of 2-8% after 2 years of follow up^6^–^12^. 90.1% of patients were asymptomatic in our study, while in two studies done in South Africa and one study in India reported asymptomatic patients in 62%, 71% and 82.7% respectively^2^–	^4^. Our study indicates a higher percentage of asymptomatic in comparison to above studies. This may be because the studies of South Africa were done using twice weekly regimen of directly observed therapy and Indian study also included the patients who had defaulted during the follow up. 16% patients had migrated to other areas in our study while in other study 43.3% had migrated. The migration (temporary or permanent) was mainly because of, movement to native place or in search of employment. This is a typical demographic feature of metropolitan cities where people from neighboring districts / states come to seek employment^13^. 10.7% patients could not be traced out in one of the study done in Mumbai; in present study 28.3% could not be traced. The reason for this high percentage of patients who were lost to follow up is probably due to the fact that 12.3% addresses of the patients were inappropriate in the record register. This could again be because of a major chunk of Indian slum dwellers lack appropriate fixed residential addresses, allocated by the Municipal Corporation.

At last we can conclude that RNTCP of India is effective in treating tuberculosis patients and 90% of patients were asymptomatic after 1 year of complication of their treatment in different categories.
